# Validation of the questionnaire for medical checkup of old-old (QMCOO) score cutoff to diagnose frailty

**DOI:** 10.1186/s12877-023-03885-3

**Published:** 2023-03-21

**Authors:** Mitsutaka Yakabe, Koji Shibasaki, Tatsuya Hosoi, Shoya Matsumoto, Kazuhiro Hoshi, Masahiro Akishita, Sumito Ogawa

**Affiliations:** 1 Department of Geriatric Medicine, Graduate School of Medicine, The University of Tokyo, 7-3-1 Hongo, Bunkyo-ku, Tokyo, 113-8655 Japan; 2 Department of Rehabilitation Medicine, Asahi Neurology and Rehabilitation Hospital, Matsudo, Japan

**Keywords:** Frailty, Prefrailty, QMCOO, KCL

## Abstract

**Background:**

Frailty is a state of increased vulnerability to poor resolution of homeostasis following a stress. Early diagnosis and intervention of frailty are essential to prevent its adverse outcomes. However, simple diagnostic criteria have not been established. The Questionnaire for Medical Checkup of Old-Old (QMCOO) is widely used for medical checkups of older adults in Japan. In our previous report, we developed a method to score the QMCOO and showed that frailty can be diagnosed with the highest accuracy when the score cutoff was set at 3/4 points. We aimed to validate the criteria in a larger cohort.

**Methods:**

Participants aged 65 years or over were recruited in the western region of Japan. They answered all the items of the Kihon Checklist (KCL) and the QMCOO. Based on the KCL score, they were diagnosed as robust (3 or lower), prefrail (4 to 7), or frail (8 or over). Then we tested the effectiveness to diagnose frailty using the QMCOO cutoff of 3/4 points. We also aimed to determine the score cutoff to separate robust and prefrail.

**Results:**

7,605 participants (3,458 males and 4,147 females, age 77.4 ± 6.9 years) were recruited. 3,665 participants were diagnosed as robust, 2,448 were prefrail, and 1,492 were frail based on the KCL score. The diagnosis of frailty had a sensitivity of 84.0%, specificity of 82.5%, and accuracy of 82.8% with a QMCOO score cutoff of 3/4 points, suggesting its validity. To separate robust and prefrail, both the accuracy and the Youden index were the highest with the QMCOO cutoff of 2/3 points (sensitivity, specificity, and accuracy were 63.9%, 83.4%, and 75.6%, respectively). All the questions of the QMCOO except Q12 (about smoking) were significantly related to prefrailty status after a logistic regression analysis.

**Conclusion:**

Diagnosis of frailty using the QMCOO score cutoff of 3/4 points was validated. Prefrailty could be diagnosed using the score cutoff of 2/3 points.

## Backgrounds

Frailty is a state of increased vulnerability to poor resolution of homeostasis following a stress. Frailty is commonly observed in older adults, supposed to be a disorder of multiple interrelated physiological systems due to an accelerated decline in physiological reserve with aging [1]. Frailty increases adverse outcomes including falls, disability, hospitalization, and mortality [1]. As the world population ages, frailty is an urgent issue.

Exercise-based interventions could delay or improve frailty [2–6], and multicomponent exercise could be especially effective [7, 8]. Early diagnosis is essential to intervene in frail patients and reduce adverse events.

The Cardiovascular Health Study (CHS) criteria by Fried et al. define frailty as having three or more of the following phenotypes: unintentional weight loss, self-reported exhaustion, weakness, slow walking speed, and low physical activity [9]. In a Japanese version of the CHS (J-CHS) criteria, the phenotypes are (i) Shrinking: “Have you unintentionally lost 2 or more kg in the past 6 months?” (yes = 1); (ii) Weakness: grip strength < 28 kg in men or < 18 kg in women (yes = 1); (iii) “In the past 2 weeks, have you felt tired without a reason?” (yes = 1); (iv) Gait speed < 1.0 m/s (yes = 1); and (v) “Do you engage in moderate levels of physical exercise or sports aimed at health?” and “Do you engage in low levels of physical exercise aimed at health?” (no to both questions = 1): frailty, prefrailty and robust were defined as having 3–5, 1–2, and 0 points, respectively [10]. The criteria are supposed to be the standard but require a grip strength tester and a 4–6 m course to measure grip strength and walking speed, consuming time to diagnose.

Diagnosis of frailty using questionnaires has been attempted. One is the Kihon Checklist (KCL), a self-reported questionnaire consisting of 25 items to screen the health and life status of older adults. The English version has been established, and all the items are described elsewhere [11]. When a KCL score of 4 to 7 points is diagnosed as prefrail and a KCL score of 8 or higher is diagnosed as frail, the best sensitivity and specificity are achieved, and the usefulness of KCL has been validated based on the frailty status diagnosed by the J-CHS criteria [12]. However, it takes time to complete the 25 items.

The Questionnaire for Medical Checkup of Old-Old (QMCOO) was established by the Ministry of Health, Labour and Welfare in Japan and has been officially used in the medical checkup of older adults in Japan. The QMCOO is self-reported by older adults. The QMCOO is aimed to assess the general health status of older adults, having 15 questions about 10 domains: health condition, mental health, eating behavior, oral function, body weight loss, physical function and falls, cognitive function, smoking, social participation, and social support. All the items of the QMCOO are described elsewhere [13]. It has been decided that the QMCOO will be used as a platform for frailty checkups for older adults in Japan. However, the QMCOO is not intended to diagnose frailty and no diagnostic criteria using the QMCOO have been established.

The QMCOO has seven questions in common with the KCL (Q4: Do you have any difficulties eating tough foods compared to 6 months ago?; Q5: Have you choked on your tea or soup recently?; Q6: Have you lost 2 kg or more in the past 6 months?; Q8: Have you experienced a fall in the past year?; Q10: Do your family or your friends point out your memory loss? e.g. “You ask the same question over and over again.”; Q11: Do you find yourself not knowing today’s date?; Q13: Do you go out at least once a week?). The QMCOO has several other questions that are not identical but similar to those in the KCL. The QMCOO has fewer items than the KCL, taking less time and burden to complete for older adults. Since the usefulness of the KCL in diagnosing frailty has been validated, the QMCOO could be used to assess frailty, but the evidence is currently insufficient.

In a previous cross-sectional study, we diagnosed frailty in community-dwelling older adults using the QMCOO. The cutoff value of 3/4 points was determined to maximize the Youden index; sensitivity, specificity, and accuracy were 76.3%, 88.1%, and 86.1%, respectively [14]. However, the number of participants in the study was 223, which is relatively small. To diagnose frailty at the same time as medical checkups using QMCOO would be useful for early intervention, and the cutoff should be validated in another larger cohort for its widespread use. In the present study, therefore, we regarded the participants as the derivation cohort and aimed to validate the cutoff in a newly established validation cohort, establish the QMCOO as a screening tool, and increase options for diagnosing frailty.

We also diagnosed robust and prefrail based on the KCL score and attempted to determine the cutoff for diagnosing prefrail using the QMCOO.

## Methods

### Study design and the participants

This is a cross-sectional study of community-dwelling older adults. Participants were recruited in the western region of Japan: Yonago City (Tottori Prefecture), Kurayoshi City (Tottori Prefecture), Masuda City (Shimane Prefecture), and Taka Town (Hyogo Prefecture). Candidate participants were those aged 65 or over who had not been certified as requiring support or care by the long-term care insurance. We mailed the candidates a paper survey that included all of the QMCOO and KCL items, and participants answered all of them and returned them. Those who had participated in our previous study [14] were excluded.

### The QMCOO and scoring

The scoring of the QMCOO was conducted as in the previous study [14]. Each question was scored as 0 or 1, and the total was the score (0–15).

### The KCL-based frailty evaluation

Each question of the KCL was scored as 0 or 1, and the total was used as the score (0–25). Based on the previous study [12], a score of 8 or higher was diagnosed as frail, a score of 4 to 7 as prefrail, and a score of 3 or lower as robust.

### Validation of the QMCOO cutoff of 3/4 points

The group of 223 participants analyzed in our previous report [13] was regarded as the derivation cohort. The group of those who agreed to participate in the present study was set as the validation cohort. The QMCOO cutoff of 3/4 points in our previous report was adopted to the validation cohort, then sensitivity, specificity, and accuracy were calculated. They were also calculated for the “75 years old or over,“ “74 years old or under,“ “males,“ and “females” groups.

### The relationship between body weight and the frailty status

We divided the participants into three groups based on the body mass index (BMI): “lean” (BMI < 18.5 kg/m^2^), “standard” (18.5 ≤ BMI < 25.0 kg/m^2^), and “obese” (BMI ≥ 25.0 kg/m^2^). Then we examined the relationship between body weight and the frailty status diagnosed by the QMCOO score. The ratio of frailty was also compared in the male and female groups. Furthermore, the participants were divided into three age groups (74 or under, 75–84, and 85 or over), then the ratio of frailty was compared in the age groups. A logistic regression analysis was performed to examine the relationship between BMI and the frailty status.

### Setting a new cutoff for diagnosing prefrail

Robust (the KCL score is three or less) and prefrail (the KCL score is 4–7) participants were extracted from the validation cohort. The cutoff score of the QMCOO for diagnosing prefrail was determined using a receiver operating characteristic (ROC) curve. The point that maximized the Youden index was adopted as the cutoff. Subgroups of age and sex were also tested for QMCOO cutoff values. A logistic regression analysis was performed to examine which of the QMCOO items determined the prefrailty status.

### Statistical analysis

A t-test was used to compare the means of two groups, and a one-way analysis of variance (ANOVA) was used to compare the means of multiple groups. Comparisons of proportions were made with a chi-square test. The Pearson test was used to calculate and test the correlation coefficient between KCL and QMCOO.

Logistic regression analysis was used to analyze the factors that affect frailty or prefrailty status. To examine the relationship between body weight and the frailty status, age, sex, and BMI were the explanatory variables, and the frailty status was the outcome. To examine which of the QMCOO items determine the prefrailty status, age, sex, BMI, and QMCOO items were the explanatory variables, and the prefrailty status was the outcome.

*P*-values < 0.05 were considered significant. All the statistical analyses were performed using R 3.3.3 software (R Foundation for Statistical Computing, Vienna, Austria).

## Results

### Validation of the QMCOO cutoff of 3/4 points

The validation cohort consisted of 7,605 people that agreed to participate and were recruited for the present study. The cohort consisted of 3,458 males and 4,147 females, and the sex ratio did not significantly differ from the derivation cohort of 103 males and 120 females (*p* = 0.900). The average age was 76.3 ± 6.9 years old in the validation cohort and significantly different from 77.4 ± 6.9 years old in the derivation cohort (*p* = 0.018). The average KCL score was 4.6 ± 3.9 in the validation cohort, and 4.2 ± 3.6 in the derivation cohort (*p* = 0.08). The average QMCOO score was 2.7 ± 2.1 in the validation cohort and 2.4 ± 2.1 in the derivation cohort (*p* = 0.012).

The characteristics of the validation cohort are shown in Table 1. Based on the KCL score, 3,665 participants were diagnosed as robust, 2,448 were prefrail, and 1,492 were frail. The average age was higher in the order of the frail group, prefrail group, and robust group, with significant differences. The average QMCOO score was also higher in the order of the frail group, prefrail group, and robust group, with significant differences. 651 of 3,458 males (18.8%) and 841 of 4,147 females (20.3%) were frail, with no significant difference in the ratio (*p* = 0.112). Height and body weight were significantly different between the robust, prefrail, and frail groups, but BMI was not.

**Table 1:** The characteristics of the validation cohort

	Robust (n = 3,665)	Prefrail (n = 2,448)	Frail (n = 1,492)	*p*-value
Sex(M/F)	1,725/1,940	1,082/1,366	651/841	0.025
Age	74.5+-6.1	77.0+-6.9	79.6+-7.2	< 0.001
QMCOO score	1.4+-1.2	3.1+-1.5	5.5+-2.1	< 0.001
Height (cm)	158.8+-8.7	157.1+-9.0	156.1+-9.5	< 0.001
Body weight (kg)	57.1+-10.0	56.0+-10.3	54.7+-11.3	< 0.001
BMI	22.6+-2.9	22.6+-3.1	22.4+-3.6	0.060

**Fig. 1:**
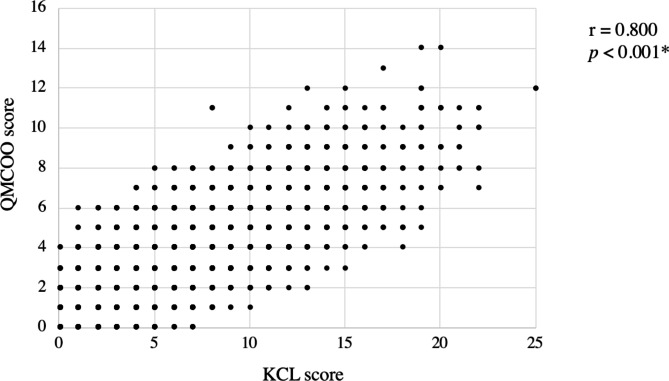
The correlation between the KCL score and the QMCOO score

The correlation coefficient between the KCL score and QMCOO score in the validation cohort was 0.800, which was significant (*p* < 0.001) (Fig. 1). In the derivation cohort, the diagnosis of frailty had a sensitivity of 76.3%, specificity of 88.1%, and accuracy of 86.1% with a cutoff of 3/4 points [13]. In all the participants in the validation cohort, sensitivity was 84.0%, specificity was 82.5%, and accuracy was 82.8%. Sensitivity, specificity, and accuracy were also good for the “75 years old or over,“ “74 years old or under,“ “males,“ and “females” groups (Table 2).

**Table 2:** Validation of diagnosing frailty by the 3/4 cutoff score of QMCOO.

	Sensitivity	Specificity	Accuracy
Total	84.0%	82.5%	82.8%
75 years old or over	84.1%	81.9%	82.5%
74 years old or under	84.0%	83.1%	83.2%
Males	86.5%	78.8%	80.2%
Females	82.2%	85.7%	85.0%

### The relationship between body weight and the frailty status

**Fig. 2:**
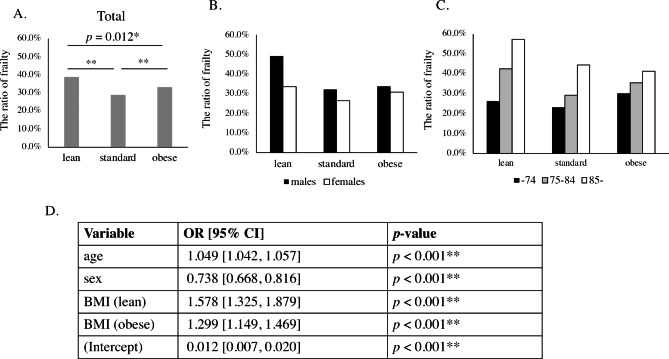
**The relationship between BMI and frailty status** Frailty was diagnosed by the QMCOO cutoff of 4 scores or over. The participants into three groups based on the body mass index (BMI): “lean” (BMI < 18.5 kg/m^2^), “standard” (18.5 ≤ BMI < 25.0 kg/m^2^), and “obese” (BMI ≥ 25.0 kg/m^2^). (A) The ratio of the frailty of lean, standard, and obese groups in total participants. (B) The ratio when the participants were divided into males and females. (C) The ratio when the participants were divided into three groups of “74 or under”, ”75–84”, and ”85 or over” (D) Logistic regression analysis to evaluate factors on frailty

In total participants, the ratio of frailty was significantly higher in the lean group and the obese group than in the standard group (Fig. 2A). When analyzed by sex, the ratio of frailty was lower in women than in men in all the groups: lean, standard, and obese (Fig. 2B). The rate of frailty was higher with age in all groups (Fig. 2C). To examine the effects of each factor on frailty status, we performed a logistic regression analysis. Age was a continuous variable, sex was a qualitative variable, and “lean” and “obese” in BMI were converted to dummy variables. After logistic regression analysis, age, sex, and BMI (both “lean” and “obese”) still significantly affected the frailty status (Fig. 2D). The variance inflation factors (VIFs) of all the explanatory variables were below 2.0, suggesting that they did not have statistical collinearity.

### Setting a new cutoff for diagnosing prefrail

**Fig. 3:**
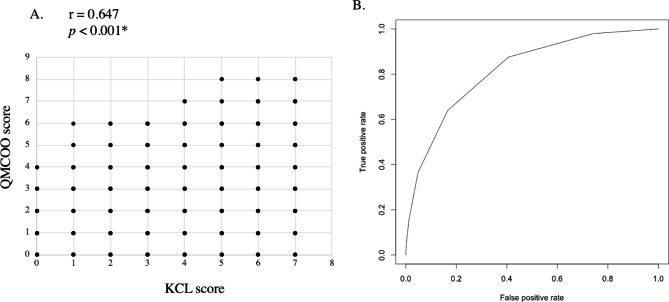
**The ROC curve and cutoff for the diagnosis of prefrailty** A. Correlation between KCL score and QMCOO score in the non-frail participants. *p* < 0.001 is considered significant B. The ROC curve was used to determine the cutoff for a diagnosis of frailty according to the QMCOO score

Robust (the KCL score is three or less, n = 3,665) and prefrail (the KCL score is 4–7, n = 2,448) participants were extracted from the validation cohort (n = 6,113 in total). The KCL score and the QMCOO score showed a significant positive correlation (Fig. 3A). The area under the curve was 0.818 (Fig. 3B). When the QMCOO cutoff was set 2/3 points, both the Youden index and the accuracy were the highest (sensitivity, specificity, and accuracy were 63.9%, 83.4%, and 75.6%, respectively). For the subgroups aged 74 or under, aged 75 or over, and males, the accuracy and the Youden index were also the highest when the cutoff was set to 2/3. However, in the subgroup of females, the accuracy was highest when the cutoff was set at 2/3, and the Youden index was highest when the cutoff was set at 1/2.

We determined the cutoff as 2/3 points and assessed its validity. The kappa statistics was 0.483 (*p* < 0.001), suggesting that those diagnosed as prefrail by the KCL tend to be diagnosed as prefrail by the QMCOO and that the cutoff has moderate reliability.

We examined which of the QMCOO questionnaire was related to the prefrailty status. Odds ratios for prefrailty in 1-score compared with 0-score are shown in Table 3. The odds ratios were significantly > 1.0 in all the questions except for Q12 (“Do you smoke?”). Then we performed a logistic regression analysis to evaluate the factors determining prefrailty. Age, sex, BMI, and scores of the questions except for Q12 were set as the explanatory variables. Low BMI was significantly related to increased risk of prefrailty, but high BMI was not (Table 4). All the other variables significantly affected the diagnosis of prefrailty. The VIFs of these variables were below 2.0.

**Table 3:** The odds ratio for prefrailty by each QMCOO question

Question	Odds ratio	Percentage of those obtaining 1-score				
		Total (n = 6,113)	>=75 years old (n = 2,985)	<=74 years old (n = 3,128)	Male (n = 2,807)	Female (n = 3,306)
Q1	5.30*	6.31%	6.97%	5.69%	7.37%	5.41%
Q2	3.53*	7.84%	6.20%	9.40%	9.16%	6.72%
Q3	1.82*	4.74%	2.91%	6.49%	5.88%	3.78%
Q4	3.78*	18.81%	21.44%	16.30%	19.67%	18.09%
Q5	3.38*	17.86%	18.99%	16.78%	18.03%	17.73%
Q6	3.27*	9.28%	8.81%	9.72%	10.30%	8.41%
Q7	4.11*	46.56%	57.45%	36.16%	45.71%	47.28%
Q8	3.52*	16.83%	18.26%	15.47%	16.99%	16.70%
Q9	1.87*	38.41%	36.92%	39.83%	37.26%	39.38%
Q10	5.07*	7.17%	7.71%	6.65%	7.73%	6.68%
Q11	3.38*	14.43%	16.78%	12.18%	15.46%	13.55%
Q12	1.00 (p = 0.989)	8.74%	4.92%	12.37%	15.96%	2.60%
Q13	2.84*	3.52%	4.32%	2.75%	2.92%	4.02%
Q14	3.57*	2.44%	1.94%	2.91%	3.85%	1.24%
Q15	2.45*	3.47%	3.28%	3.64%	4.85%	2.30%

Odds ratios for prefrailty in 1-score compared with 0-score are shown. *p < 0.05 is considered significant

**Table 4:** Logistic regression analysis to evaluate factors on prefrailty

Variable	OR [95% CI]	*p*-value
age	1.052 [1.041, 1.063]	< 0.001*
sex	1.343 [1.176, 1.534]	< 0.001*
BMI (lean)	2.195 [1.705, 2.825]	< 0.001*
BMI (obese)	1.082 [0.919, 1.274]	0.341
Q1	3.156 [2.375, 4.195]	< 0.001*
Q2	2.738 [2.135, 3.512]	< 0.001*
Q3	1.748 [1.293, 2.364]	< 0.001*
Q4	3.953 [3.355, 4.658]	< 0.001*
Q5	3.626 [3.067, 4.286]	< 0.001*
Q6	3.956 [3.166, 4.943]	< 0.001*
Q7	2.993 [2.613, 3.427]	< 0.001*
Q8	3.545 [2.991, 4.202]	< 0.001*
Q9	1.658 [1.451, 1.896]	< 0.001*
Q10	5.174 [3.971, 6.742]	< 0.001*
Q11	3.326 [2.773, 3.989]	< 0.001*
Q13	4.874 [3.459, 6.870]	< 0.001*
Q14	3.820 [2.430, 6.003]	< 0.001*
Q15	1.619 [1.102, 2.379]	0.014*
(Intercept)	0.001 [0.001, 0.003]	< 0.001*

## Discussion

In the present study, we have demonstrated the validity of the diagnosis of frailty with a QMCOO score cutoff of 3/4 points.

The QMCOO includes a question about weight loss (Q6), but unlike the KCL, does not include BMI itself. When considering the relationship between BMI and physical function, sarcopenia should also be considered. Sarcopenia is a progressive and generalized skeletal muscle disorder typically observed in older adults, requiring lower appendicular muscle mass or lower muscle quality for diagnosis in the EWGSOP2 criteria [15]. Lower BMI was related to an increased risk of sarcopenia [16]. Sarcopenia is associated with functional decline and increased risk of frailty [17]. Thus it is plausible that lower BMI was associated with frailty in the present study, but higher BMI was also associated with frailty (Fig. 2). The relationship between BMI and the prevalence of frailty is suggested to form a U-shape. A study of British people showed that the BMI range of the lowest prevalence of frailty was 25.0-29.9 kg/m^2^ [18], but in another study, the range was 18.5–24.9 kg/m^2^ [19]. In a study of community-dwelling Japanese older people, the prevalence of frailty was lowest in the BMI range of 21.4–25.7 kg/m^2^ [20]. In the present study, the prevalence of frailty diagnosed using the QMCOO was the lowest in the BMI range of 18.5–25 kg/m^2^, compatible with previous findings.

Prefrailty was significantly associated with lower BMI but not with higher BMI (Table 4). The score of Q6 (weight loss) affected the prefrailty status after a logistic regression analysis. Therefore, the experience of body weight loss itself might be the risk of prefrailty, independently of BMI. This suggests that maintaining an appropriate BMI might be important to prevent prefrailty, thus avoiding frailty. However, as little is known about the background of the participants in the study, some diseases (e.g., malignancy, infections, etc.) other than natural aging could result in body weight loss, developing prefrailty or frailty.

We also demonstrated that a QMCOO score cutoff of 2/3 points might help diagnose prefrailty. By picking up patients with a QMCOO score of 3 or more, it might be possible to diagnose and intervene in frailty at an earlier stage. All questions except Q12 (smoking) were significantly associated with the diagnosis (Table 3). In our previous report including 223 participants, only Q1, Q6, Q7, Q10, and Q11 were related to the diagnosis of frailty [14]. In the present study, the number of participants (n = 6,113) might have sufficient statistical power.

Identifying aspects of frailty and prefrailty is essential to establish their diagnostic methods. Q1 (subjective health status) and Q2 (subjective satisfaction with daily life) are unique to the QMCOO, not included in the J-CHS, the KCL, and the five-item frailty screening index [21]. The scores of both questions were significantly related to prefrailty status after the multiple linear regression analysis (Table 4). These straightforward questions about subjective health status and satisfaction could be considered to be included in a new questionnaire. Furthermore, other QMCOO items, such as Q6 (body weight loss), Q7 (loss of walking speed), and Q13 (habits of walking), significantly affected prefrailty and frailty status. By picking appropriate items from the QMCOO, a new frailty questionnaire could be developed.

An important limitation of our study is that we had very limited information about the participants. We used only data about the participants’ age, sex, height, body weight, and answers to the questionnaires, but other data were missing. We included age, sex, and BMI as the explanatory variables in the logistic regression analysis but could not consider other confounding factors that might affect the frailty/prefrailty status. Only those who had not been certified as requiring support or care by the long-term care insurance were recruited. However, older adults in general tend to have multiple comorbidities even if they are independent. As stated earlier, sarcopenia and diseases could cause body weight loss and lower gait speed, which are characteristics of frailty/prefrailty. Furthermore, other factors (medication, past medical history, protein and calorie intake, exercise habits, social status, etc.) should also be considered as explanatory variables in the analysis.

Since the QMCOO will be used as a platform for frailty checkups for older adults in Japan, diagnosing frailty at the same time as medical checkups can contribute to medical care for older adults. The QMCOO could be used for screening, then older adults would be formally diagnosed as frail according to the J-CHS, which is supposed to be the standard. However, the present study has limitations. To establish the QMCOO as a diagnostic tool, further studies are needed on older adults with more information about their background. In addition, the KCL was used instead of the J-CHS criteria for the diagnosis of frail and prefrail in the present study, but further research using the J-CHS is needed. Furthermore, this is a cross-sectional study in four limited areas, and the QMCOO should be validated in other regions. Thus by accumulating evidence, the QMCOO might contribute to early diagnosis and intervention of frailty and prefrailty in the future.

## Conclusion

Diagnosis of frailty using the QMCOO score cutoff of 3/4 points was validated. Prefrailty could be diagnosed using the QMCOO score cutoff of 2/3 points. The QMCOO could be a screening tool for early diagnosis of frailty.

## Data Availability

The analyzed datasets are available from the corresponding author upon reasonable request.

## Abbreviations

QMCOO: Questionnaire for Medical Checkup of Old-Old
KCL: Kihon Checklist
CHS: Cardiovascular Health Study
J-CHS: Japanese version of the CHS
BMI: body mass index
ROC: receiver operating characteristic
ANOVA: analysis of variance
VIFs: variance inflation factors
EWGSOP: European Working Group on Sarcopenia in Older People
